# Cation Tuning of Polaron Barriers in Layered Perovskites for Optical Spin Lifetime Control

**DOI:** 10.1021/acsenergylett.5c01236

**Published:** 2025-08-31

**Authors:** Valentino Romano, Martin Hörmann, Anna Stadlbauer, Edoardo Mosconi, Luca Gregori, Filippo De Angelis, Felix Deschler, Giulio Cerullo, Franco V. A. Camargo

**Affiliations:** † Physics Department, 18981 Politecnico di Milano, Piazza Leonardo da Vinci 32, 20133, Milano, Italy; ‡ 529704 Walter-Schottky-Institute, Physics Department, Technical University Munich, Munich 85748, Germany; § Institute for Physical Chemistry, 9144 Heidelberg University, Im Neuenheimer Feld 229, 69120 Heidelberg, Germany; ∥ Computational Laboratory for Hybrid/Organic Photovoltaics (CLHYO), Istituto CNR di Scienze e Tecnologie Chimiche “Giulio Natta” (CNR-SCITEC), Via Elce di Sotto 8, 06123 Perugia, Italy; ⊥ Department of Chemistry, Biology and Biotechnology, 9309 University of Perugia, Perugia 06123, Italy; # SKKU Institute of Energy Science and Technology (SIEST), Sungkyunkwan University, Suwon 440-746, South Korea; ○ Istituto di Fotonica e Nanotecnologie, 9327 Consiglio Nazionale delle Ricerche, Piazza Leonardo da Vinci 32, 20133, Milano, Italy

## Abstract

Layered metal-halide perovskites (L-MHPs) form self-assembled quantum wells with strongly bound excitons and electron–phonon interactions that promote polaron formation. Due to spin–orbit coupling and Rashba-type spin-splitting of the electronic bands, spin-polarized excitons can be photoexcited with circularly polarized light, making these materials promising in opto-spintronics. Recently, we have shown that photoexcitation with excess energy extends spin-lifetimes in (BA)_2_FAPb_2_I_7_ by over 2 orders of magnitude compared to resonant excitation and attributed this to polaron formation. Here, we study spin-lifetimes in L-MHPs with different A-site cations: (Hexa)_2_MAPb_2_I_7_, (Hexa)_2_FAPb_2_I_7_, (Hexa)_2_CsPb_2_I_7_ (Hexa: hexylammonium, MA: methylammonium, FA: formamidinium, Cs: cesium). We find that all studied materials exhibit vastly extended spin-lifetimes under excess-energy excitation, but that the polaron formation barrier is reduced with increasing polarity of the A-site cations. First-principles calculations show that (Hexa)_2_MAPb_2_I_7_ has the most stable polarons and (Hexa)_2_CsPb_2_I_7_, the least. Our findings demonstrate tuning of optically controlled exciton spin-lifetimes in L-MHPs through composition engineering, providing a pathway toward optimized materials for spintronics.

## Graphical abstract

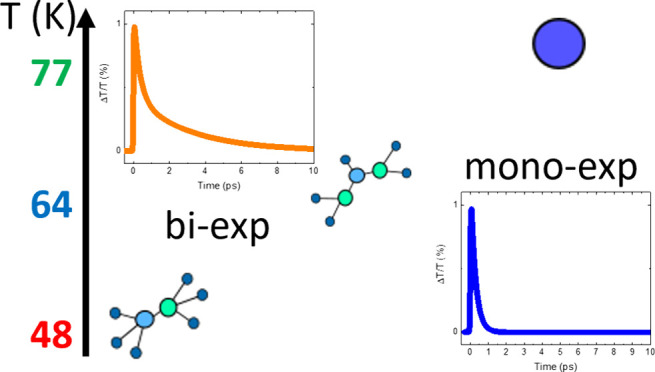

Layered metal-halide perovskites (L-MHPs) are self-assembled quasi-2D quantum-well structures characterized by an alternating sequence of the metal-halide octahedra and organic cation molecules.^1,2^ They have the general formula R_2_A_*n*–1_B_*n*_X_3*n*+1_, where R is a large organic cation, A is a small monovalent cation, B is a divalent metal species, X is a halide, and *n* is the number of inorganic layers sandwiched between organic layers. The resulting quantum confinement leads the charge carriers to form excitons with binding energies of up to 500 meV, which are tunable by the thickness and the chemical composition of the inorganic layer.^3–8^ Moreover, the inherently soft and polar nature of the perovskite lattice is responsible for dynamic disorder and anharmonicity^9^ which, in turn, influence interactions between charge carriers and phonons. This coupling is known to cause the formation of exciton polarons, i.e., excitons dressed by the coupling with the lattice, in bulk as well as in layered perovskites,^10–12^ making the latter an intriguing platform for fundamental studies on the interplay between polaronic and excitonic features.

The presence of heavy atoms endows perovskites with large spin–orbit coupling (SOC), i.e., a shift in energy of the electronic bands and coupling between spin and angular momentum of electronic states. In particular, in bulk MAPbI_3_ SOC splits the conduction band (CB, where *L* = 1) into a lower (*J* = 1/2, responsible for the optical band gap of the material) and an upper (*J* = 3/2) band, while the valence band (VB) maximum (where *L* = 0) is unaffected.^13^ For near bandgap excitation, only the bottom of the CB and the top of the VB are populated which, in general, are spin-degenerate. However, in crystal structures lacking an inversion symmetry point SOC lifts the spin degeneracy, causing a shift in momentum of the electronic bands, a phenomenon known as Rashba splitting (Figure 1a). As a result, in MHPs spin-polarized charge carriers can be generated by illumination with circularly polarized light (Figure 1a). Interestingly, the spin–orbit interactions accelerate the relaxation of hot carriers,^14^ and the spin-polarization in MHPs reaches values as high as twice the one observed in conventional III–V and II–VI semiconductors.^15–17^ Thus, the potential of MHPs as materials for spintronic applications started being explored,^18–24^ with studies spanning phenomena such as chirality-induced spin selectivity^25–29^ and material properties affecting spin lifetimes.^30–36^

**Figure 1:**
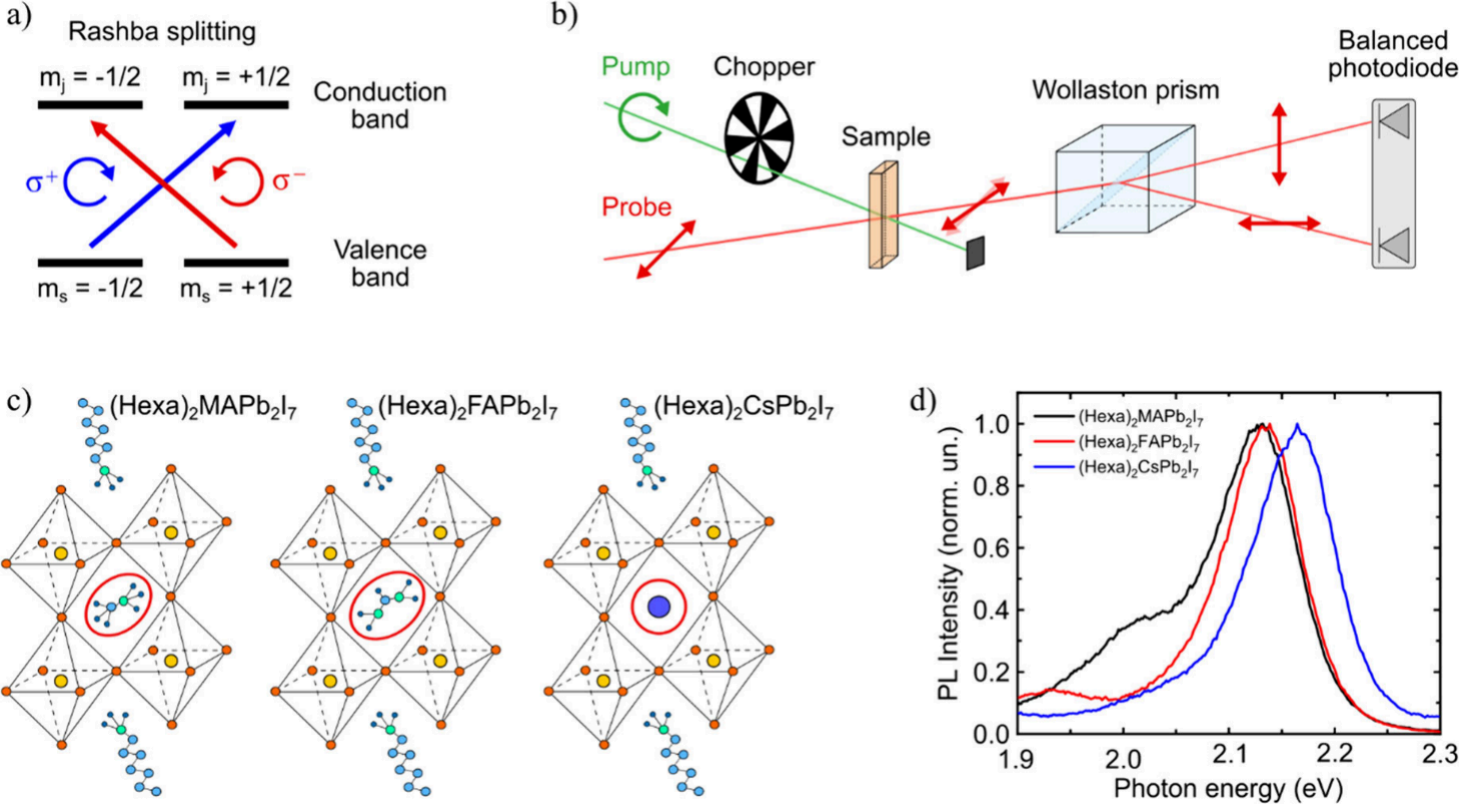
(a) Schematic representation of the effects of spin–orbit coupling and Rashba splitting on the electronic energy bands of MHPs. (b) Sketch of the setup used to perform TRFR measurements. (c) Crystal structure of the selected L-MHPs. (d) Photoluminescence spectra of the investigated samples.

Recently, some of us reported that spin lifetimes of (BA)_2_FAPb_2_I_7_ (where BA: butylammonium; FA: formamidinium) can be increased by 2 orders of magnitude upon photoexcitation with excess energy.^37^ Time-resolved Faraday rotation (TRFR) experiments upon resonant excitation of excitons showed that their spin relaxation is governed by a motional-narrowing mechanism due to precession around an effective magnetic field. In this regime spin lifetimes become shorter at low temperatures, reaching ∼0.2 ps at 77 K. At higher temperatures, the lifetimes showed biexponential kinetics, with the longer component becoming slower as the temperature was lowered, which hinted at a potential double relaxation mechanism. This was confirmed by low-temperature photoexcitation at with excess photon energy, which revealed progressively longer-lived spin polarization signals as the excess photon energy of the pump was increased, going from ∼0.2 ps to beyond 50 ps. In all cases, a fast ∼0.2 ps component remained present. Since hot-carrier cooling in L-MHPs takes place on a similar time scale as spin relaxation of the excitons,^38–40^ the coexistence of short and long lifetimes at 77 K suggests that this unusual behavior is due to the temperature dependent formation of a new state with a different spin relaxation mechanism. We attributed this peculiar behavior to a thermally activated exciton-polaron formation, whereby the modified exchange interaction in polarons with respect to excitons is responsible for the different spin relaxation mechanism.

Here, we use femtosecond TRFR (Figure 1b) to investigate the polaron formation mechanism in a family of (*n* = 2) L-MHPs by systematically varying the A-site cation from methylammonium ((Hexa)_2_MAPb_2_I_7_) to formamidinium ((Hexa)_2_FAPb_2_I_7_) and to the inorganic cation cesium ((Hexa)_2_CsPb_2_I_7_), where Hexa is hexylammonium (Figure 1c). In all these systems we observe that optical spin lifetime control by temperature and excitation photon energy is possible and identify the barrier for polaron formation by the temperature dependence of the spin-depolarization dynamics.^37^ Moreover, in agreement with first-principles numerical simulations, we find that the polaron formation barrier follows an opposite trend to the polarity of the A cation, suggesting that larger polarities favor polaron stabilization. These findings demonstrate the ability to control spin depolarization lifetimes by engineering the chemical composition of L-MHPs, in view of opto-spintronic applications of these materials.

All investigated thin film samples are prepared by spin-coating (see Supporting Information for an in-depth discussion of the protocol employed) and show a good phase purity, as evidenced by their photoluminescence (Figure 1d) and transient absorption characterizations (Figure S1). The spin-polarization lifetime was studied with TRFR (Figure 1b), a technique in which ultrashort circularly polarized excitation pulses are used to generate a spin polarized carrier population, which causes a transient chiro-optical refractive index difference between left and right-handed circularly polarized light. Changes in the imaginary part of the refractive index cause a difference in absorption between light of opposite helicities (circular dichroism) leading to elliptical polarization. On the other hand, changes in the real part correspond to a difference in the phase delay between light of opposite helicities (optical rotatory dispersion), resulting in a rotation of the axis of a linearly polarized probe pulse (Faraday rotation), with the rotation angle proportional to the net spin polarization in the sample. TRFR efficiently measures the latter by using a Wollaston prism to project the orthogonal polarization components of the probe pulse onto a pair of balanced photodiodes followed by a differential amplifier. If a polarization rotation due to a chiral change of the refractive index occurs, the light intensity impinging on the photodiodes is unbalanced, and the differential signal measures the Faraday rotation. The TRFR measurements (Figure 1b) were performed using circularly polarized 100 fs tunable pump pulses with a full-width-at-half-maximum bandwidth of ∼40 meV, while the probe pulses were tuned to 2.07 eV, well below the bandgap, where the dispersive TRFR line shape extends.^41^ For all the measurements, we conducted a pump fluence dependence, to exclude exciton–exciton annihilation, and selected only low fluence data where nonlinear kinetics are negligible (Figures S2 and S3 in the Supporting Information show the data from the main text at two different fluences).

Figure 2 shows the comparison between the TRFR dynamics measured at 77 K of (Hexa)_2_MAPb_2_I_7_ (Figure 2a), (Hexa)_2_FAPb_2_I_7_ (Figure 2b), and (Hexa)_2_CsPb_2_I_7_ (Figure 2c) following excitation in resonance (black curve) and with excess photon energy (red curve) with respect to their optical bandgaps. All samples exhibit spin depolarization dynamics that are faster following resonant excitation and slower when pumped with excess photon energy, which we attribute, in agreement with our previous study,^37^ to the formation of polaronic states. We note that following 2.43 eV excitation, the Faraday signal shows a rising component in the first few hundred femtoseconds in Figure 2c. This component reflects the cooling of the hot carriers toward the bandgap promoted by carrier-phonon scattering, which is much less prominent in (Hexa)_2_CsPb_2_I_7_ (see discussion of Figure 5 below). Interestingly, for resonant excitation, we observe that the TRFR dynamics in (Hexa)_2_MAPb_2_I_7_ and (Hexa)_2_FAPb_2_I_7_ follow a biexponential trend, consisting of a strong decay on ∼200 fs time scales followed by a small signal that fully decays after several picoseconds. On the other hand, the signal in the (Hexa)_2_CsPb_2_I_7_ sample displays a purely monoexponential behavior, fully decaying in the first picosecond. This suggests that, under these conditions, formation of polaronic states in (Hexa)_2_CsPb_2_I_7_ is slower than the exciton spin lifetime, whereas in (Hexa)_2_MAPb_2_I_7_ and (Hexa)_2_FAPb_2_I_7_ some polaron formation can take place before all exciton spin polarization is lost.

**Figure 2:**
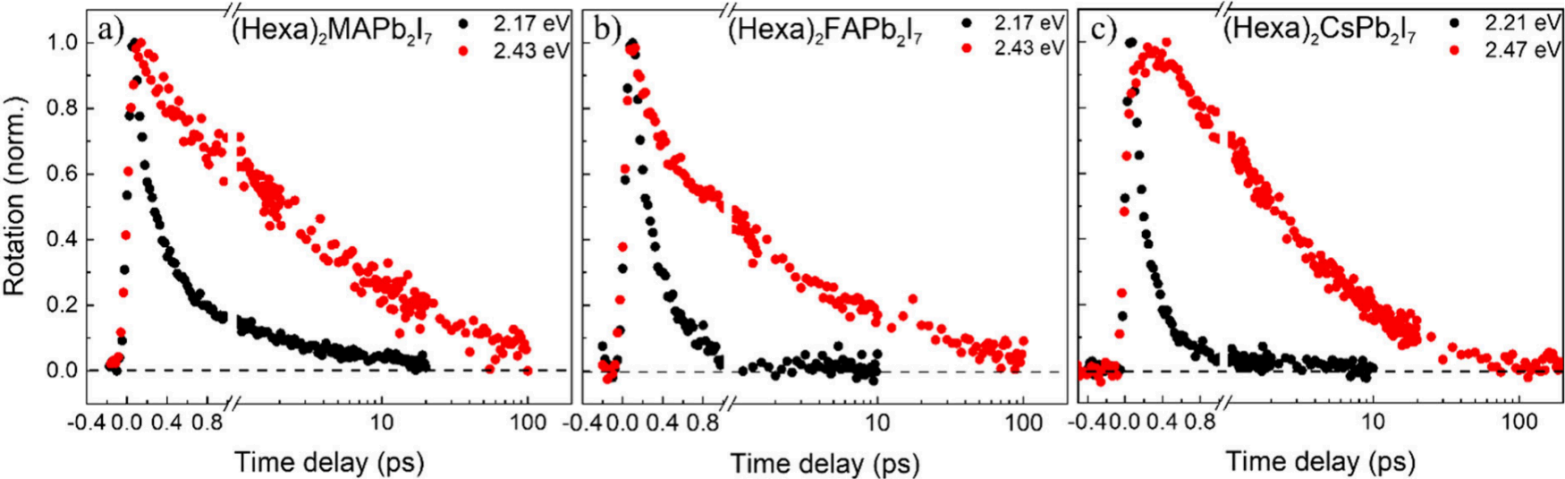
TRFR decays of (a) (Hexa)_2_MAPb_2_I_7_, (b) (Hexa)_2_FAPb_2_I_7_, (c) (Hexa)_2_CsPb_2_I_7_ measured with optical excitation close to resonance (black curves) and above resonance (red curve) with respect to the excitonic optical bandgap, at the stated photon energies. Measurements were performed at 77 K. The excitation photon energy was chosen to provide the same amount of excess energy (260 meV) with respect to the optical bandgap of each material.

To further understand spin depolarization processes in these materials, we performed temperature-dependent TRFR measurements in the range 4–300 K using resonant bandgap excitation (Figure 3). All investigated samples show longer spin lifetimes as the temperature is increased. Moreover, we note that in the very low temperature range all samples present a monoexponential spin decay with lifetime of the order of 0.2 ps, whereas at higher temperatures a multiexponential decay is observed. The fast component at low temperatures is consistent with the motional narrowing spin relaxation mechanism previously described in similar materials, which we attribute to the excitonic species. At higher temperatures, on the other hand, the polaron formation rate is increased, eventually becoming comparable to the exciton spin depolarization rate. At this point, polaron formation can take place before spin relaxation is complete, so that the TRFR kinetics are a result of both exciton and polaron spin relaxation processes as well as the equilibration time between the two species.

**Figure 3:**
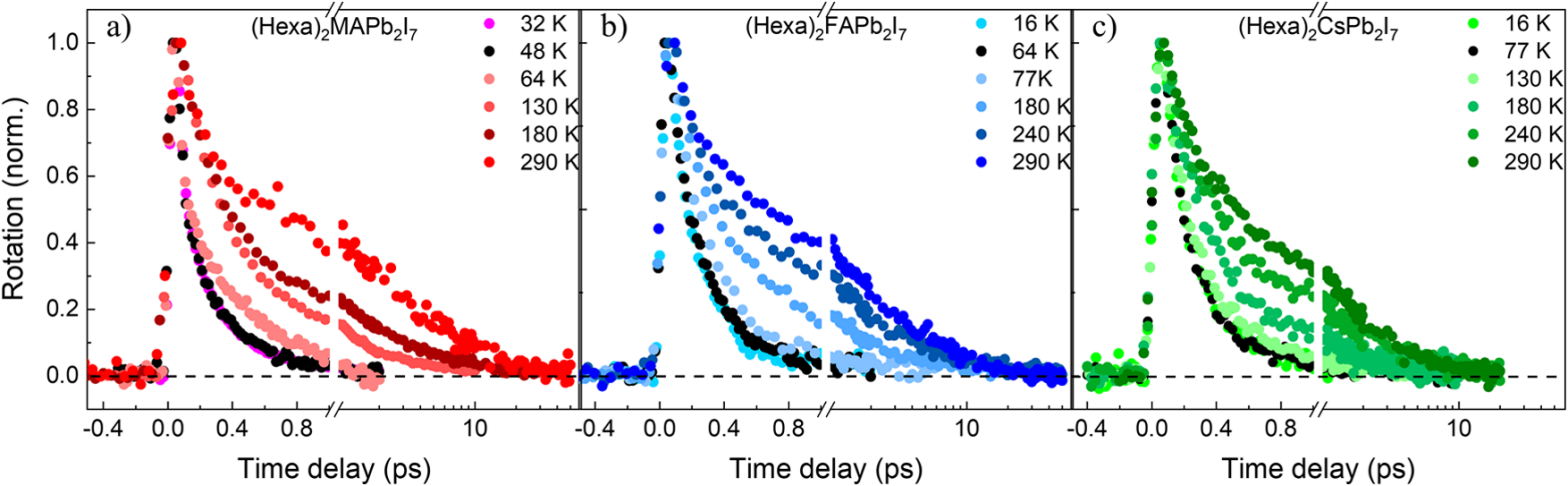
Temperature dependence of the TRFR signals with pump close to resonance with the optical bandgap, performed on (a) (Hexa)_2_MAPb_2_I_7_, (b) (Hexa)_2_FAPb_2_I_7_, and (c) (Hexa)_2_CsPb_2_I_7_. The black curves highlight the highest temperatures for which the decay is still monoexponential.

In this context, the threshold temperature between the mono- and the biexponential spin relaxation regimes can be associated with the polaron formation barrier for each sample: the higher this temperature, the more energy is required to form polarons before the exciton spin polarization is lost. Figure 3 reveals that this critical temperature is lowest for MA (∼48 K) and highest for Cs (∼130 K), while FA shows an intermediate value (∼64 K). Interestingly, these values display an opposite trend with respect to the expected dipole moment trend of the involved A-site cations, with MA more polar than FA and Cs.

Our TRFR experiments confirm that the spin depolarization dynamics changes when samples are pumped with excess photon energy, producing lattice heating from carrier cooling, and/or at sample temperatures higher than a specific threshold. We associate the origin of this phenomenon to the formation of polarons in L-MHPs that shield excitons from spin-depolarization processes. Indeed, since L-MHPs are polar materials, the Fröhlich interaction is the most effective carrier-phonon coupling pathway. Yet, since optical phonons are mostly not dispersive, their population strongly depends on temperature; i.e., below a threshold temperature there is insufficient energy to effectively generate such vibrational quanta.

To elucidate the mechanisms underlying the localization and stabilization of hole polarons in the studied systems, we conducted structural relaxation calculations in the presence of an additional positive charge, as reported in our previous work.^42^ We chose to focus on hole polarons mainly because previous studies have shown that electron polaron stabilization energies in these materials are much smaller in magnitude than hole polarons.^43^ For each system, the stabilization energy is calculated as Δ*E*_pos_ = *E*_v_ – *E*_r_, where *E*_v_ is the total energy of the supercell after the vertical injection of a positive charge into the neutral system and *E*_r_ is the total energy of the charged supercell after structural relaxation (Figure S4 shows a schematic representation of these quantities). In addition, we also evaluate the relaxation energy of the neutral system, *E*_neu_, which represents the energy required to bring the neutral system from the geometry optimized in the positively charged state to its true ground-state geometry, i.e., the geometry optimized in the neutral charge state. This quantity provides insight into the structural differences induced by the presence of a localized hole and allows us to quantify the structural response across different A-site cations. Electrostatic finite-size effects in charged periodic supercell calculations are neglected due to the large dielectric constant of the system.^44^ The calculated values are reported in Table 1, while Figure 4 illustrates the charge density difference between neutral and charged system.

**Figure 4:**
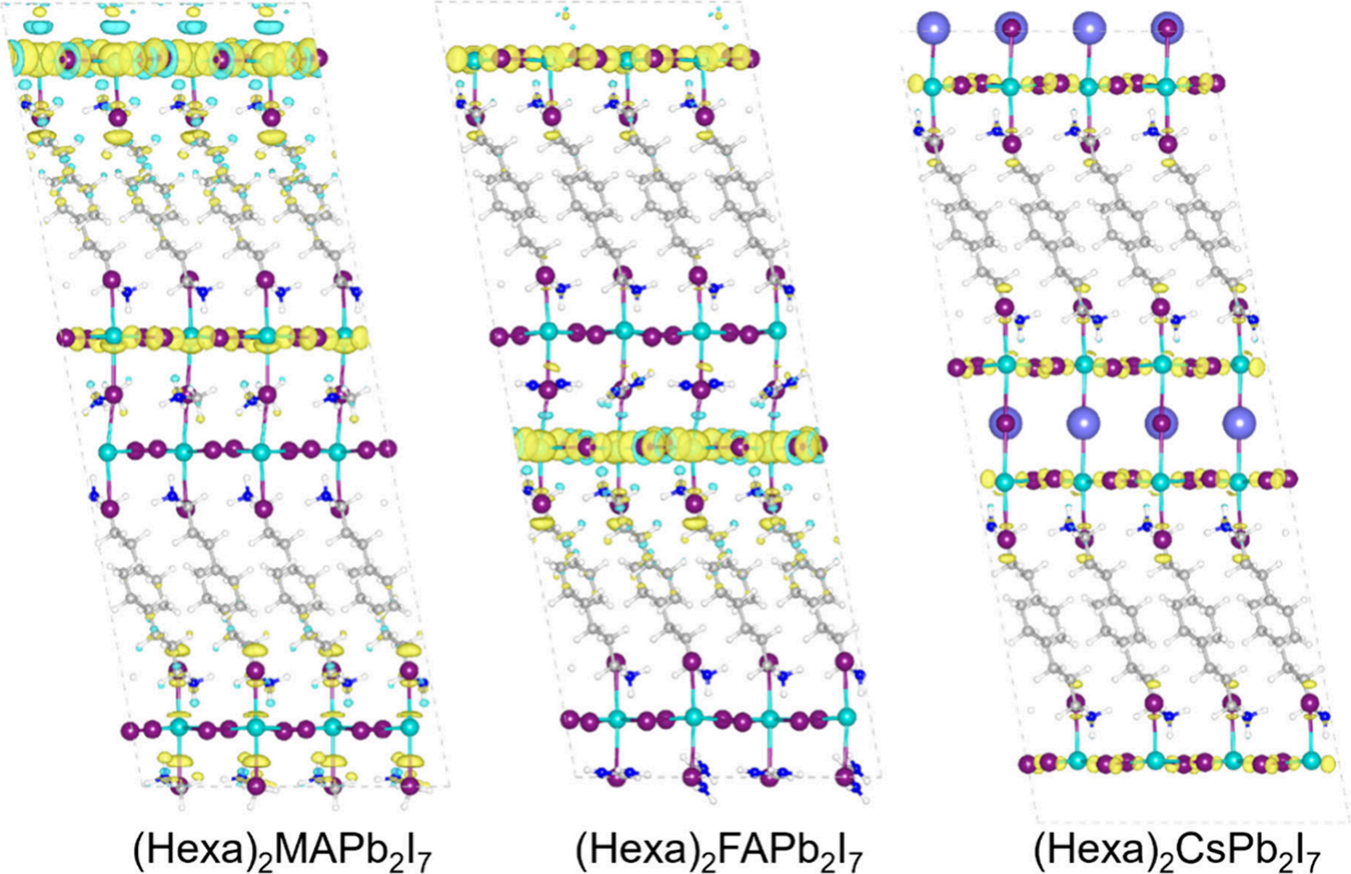
Isodensity plot for the three structures representing the charge density difference obtained as the difference between the total density of the fully relaxed positively charged system and that of the neutral system at the positive charged-state geometry, revealing a localization of the trapped charge on the inorganic framework. Color scheme: Pb, cyan; I, magenta; C, gray; N, blue; H, white. The isovalue is set to 0.0002 A^3^ for all the reported isodensities.

**Table 1:** Calculated Values of Stabilization Energies for Hole Polaron (Δ*E*_pos_) and Relaxation Energy upon Emission (Δ*E*_neu_) for the Three Different Systems at the PBE0-D3 Level of Theory

	energy (meV)	
system	Δ*E*_pos_	Δ*E*_neu_
(Hexa)_2_MAPb_2_I_7_	44	70
(Hexa)_2_FAPb_2_I_7_	29	30
(Hexa)_2_CsPb_2_I_7_	16	16

Table 1 shows a systematic trend in the stabilization energies. In particular, the stabilization energy decreases as we move from (HexA)_2_MAPb_2_I_7_ to (HexA)_2_FAPb_2_I_7_ and to (HexA)_2_CsPb_2_I_7_. This trend is particularly interesting as it correlates with the polarity of the cation residing within the 3D cage of the perovskite structure. The higher stabilization energy observed for (HexA)_2_MAPb_2_I_7_ can be attributed to the higher polarity of the MA cation, which enhances the interaction with the localized charge (hole polaron). In contrast, the lower values of the stabilization energies for (HexA)_2_FAPb_2_I_7_ and (HexA)_2_CsPb_2_I_7_ reflect the reduced polarity of FA and Cs, respectively. This correlation suggests that the polarity of the cation plays a critical role in modulating the energetics of charge localization and stabilization in these layered perovskite systems.^42,45–47^

Additional information about the coupling between charges and phonons can be obtained from transient absorption (TA) measurements via the observation of impulsive stimulated Raman scattering, which causes periodic oscillations of the transmitted probe light intensity due to modulation of the sample transmission by photoinduced coherent lattice vibrations.^48^ Figure S4 shows the differential transmission (Δ*T*/*T*) maps, as a function of probe photon energy and delay, of the three investigated samples measured at 77 K under resonant excitation conditions, using a broadband probe (1.653–2.755 eV). The data show the typical contributions observed in the TA spectra of MHPs: a positive signal at the excitonic peak, associated with the photobleaching of the excitonic transition, and the two negative bands on its sides associated with shift and broadening of the excitonic transition.

A global analysis of these data sets allows to fit the slowly varying contribution to the measured TA maps which, once subtracted from the data, yields a residual oscillatory signal, indicative of impulsive stimulated Raman scattering. Figure 5a shows the signal for (Hexa)_2_FAPb_2_I_7_, while the data for (Hexa)_2_MAPb_2_I_7_ and (Hexa)_2_CsPb_2_I_7_ are presented in Figure S5. Figure 5b shows oscillatory kinetics at two representative wavelengths for the case of (Hexa)_2_FAPb_2_I_7_: we observe a clear π phase flip of the oscillatory pattern between 2.145 and 2.175 eV probe energies which are, respectively, red- and blue-shifted with respect to the excitonic peak. This indicates that the coherent phonons are strongly coupled to the excitonic transition. To get more insight into the phonon modes modulating the excitonic transition, we computed the Fourier transform of the data shown in Figure 5a and Figure S6. The power spectra of the oscillatory signal component are reported in Figure 5c for the three samples as a function of the probe photon energy (the results are normalized with respect to the maximum of the TA traces of the corresponding material). We observe that the power spectra have a lower intensity in (Hexa)_2_MAPb_2_I_7_ (data multiplied by a factor 1.33) and (Hexa)_2_CsPb_2_I_7_ (data multiplied by a factor 4) relative to (Hexa)_2_FAPb_2_I_7_, suggesting that the hybrid compositions experience a stronger exciton–phonon coupling than the inorganic counterpart. A more quantitative comparison between the different chemical compositions is provided by integrating the power spectra over probe photon energy and wavenumber, obtaining a quantity that is proportional to the energy of the coupled vibrational modes. Since we observe contributions distributed in two main regions (18–40 cm^–1^ and 40–60 cm^–1^), we separate the calculations of the integral of these areas (the exact range is shown and reported in Figure S7). The results are given in Table 2.

**Figure 5:**
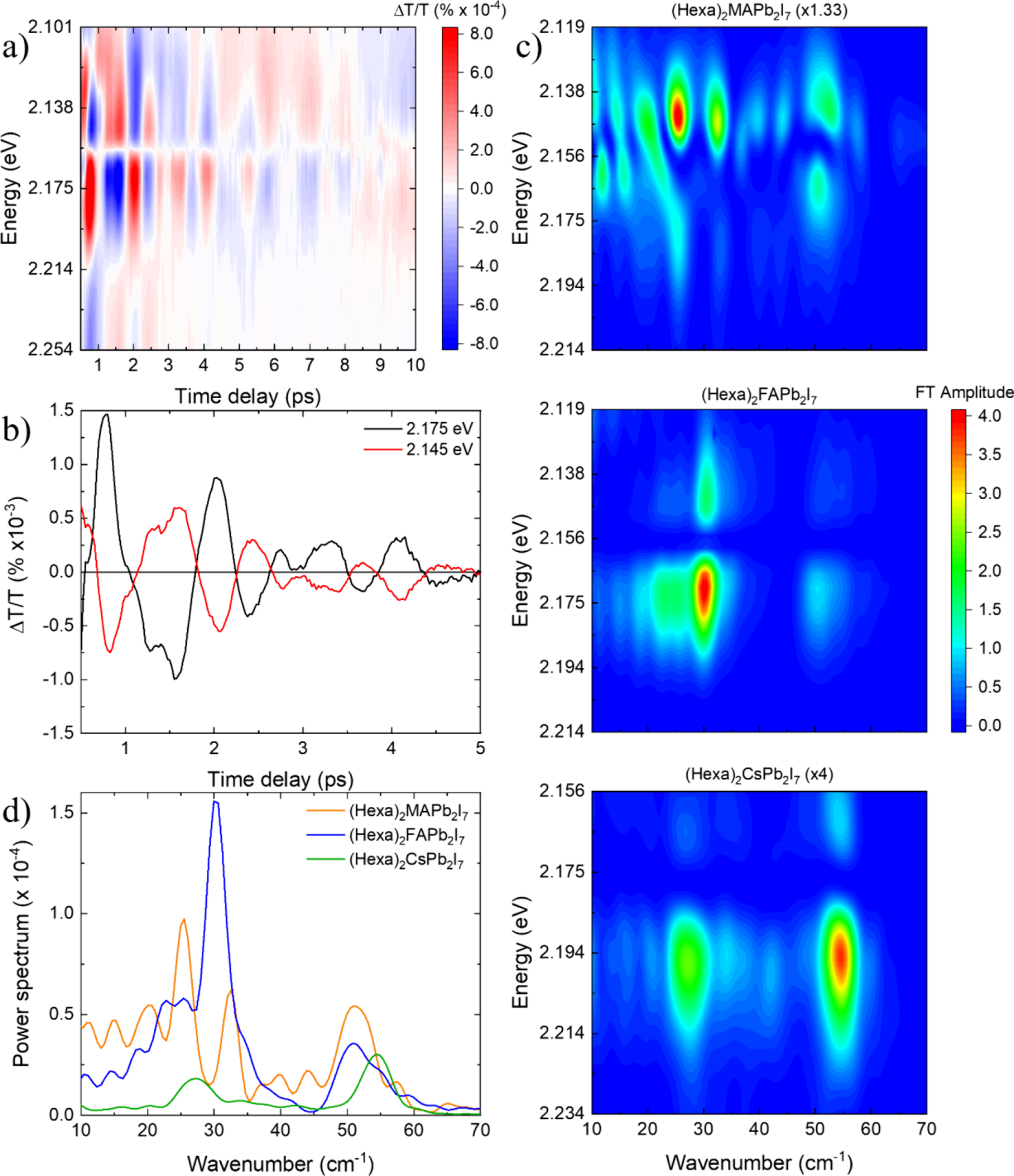
(a) Oscillatory components of the TA map of (Hexa)_2_FAPb_2_I_7_. (b) Representative coherent oscillations observed in (Hexa)_2_FAPb_2_I_7_, obtained by subtracting the fitted slowly varying component of the signal from the TA map. (c) Comparison of the power spectra maps obtained from the residuals of (Hexa)_2_MAPb_2_I_7_ (top), (Hexa)_2_FAPb_2_I_7_ (middle), and (Hexa)_2_CsPb_2_I_7_ (bottom). Data for (Hexa)_2_MAPb_2_I_7_ and (Hexa)_2_CsPb_2_I_7_ are multiplied by 1.33 and 4, respectively, to account for the differences in maximum TA signal. (d) Comparison of the power spectra of the investigated materials. Measurements were performed at 77 K and with excitation in resonance with the optical bandgaps of the samples.

**Table 2:** Integral of the Power Spectra of the Investigated Samples over the Main Raman Bands

system	18–40 cm^–1^	40–60 cm^–1^
(Hexa)_2_MAPb_2_I_7_	0.0178	0.0105
(Hexa)_2_FAPb_2_I_7_	0.0255	0.0070
(Hexa)_2_CsPb_2_I_7_	0.0043	0.0051

We observe that (Hexa)_2_MAPb_2_I_7_ and (Hexa)_2_FAPb_2_I_7_ show stronger contributions, respectively, in the range 40–60 and 18–40 cm^–1^, while (Hexa)_2_CsPb_2_I_7_ exhibits lower values in both regions. This outcome further suggests that the hybrid compositions experience a stronger exciton–phonon coupling with respect to (Hexa)_2_CsPb_2_I_7_. Finally, with the aim to get insight into the frequencies of the vibrational modes discussed, we integrate the power spectra reported in Figure 5c over the probe photon energy (2.119–2.198 eV for (Hexa)_2_MAPb_2_I_7_ and (Hexa)_2_FAPb_2_I_7_; 2.156–2.230 eV for (Hexa)_2_CsPb_2_I_7_). Figure 5d compares the integrated PS for the three materials, normalized with respect to the maximum of the corresponding TA maps.

In MHPs, in particular, in the prototypical MAPbI_3_ perovskite, it has been shown that below 200 cm^–1^ the vibrational density of states consists of 80% contributions from internal vibrations of the inorganic network (PbI_3_), while the remainder is associated with spinning, translations and librations of the MA^+^ cation.^49,50^ This is due to the heavy masses of the atoms which constitute the inorganic network (lead/tin and halogens) which result in oscillators whose normal modes have a low frequency. Thus, since L-MHPs have an inorganic framework like that of their bulk counterparts, the low-frequency region is characterized by similar phonon modes, although some shifts are expected because of the different chemical environment. In the range 40–60 cm^–1^ we observe similar vibrational features in both (Hexa)_2_MAPb_2_I_7_ and (Hexa)_2_FAPb_2_I_7_, while those in (Hexa)_2_CsPb_2_I_7_ differ both in intensity and frequency. Finally, the range 18–40 cm^–1^ shows starker differences among the three samples: the intensity, frequency, and number of modes are strongly dependent on the chemical composition. We suggest that these modes are due to translations and librations of the cage cations (MA^+^, FA^+^, Cs^+^). Irrespective of the exact identification of the involved vibrational modes, it is clear that (Hexa)_2_MAPb_2_I_7_ and (Hexa)_2_FAPb_2_I_7_ experience stronger exciton–phonon coupling with respect to (Hexa)_2_CsPb_2_I_7_, consistent with the stabilization energy trend of the excitonic polarons.

We have used femtosecond TRFR to experimentally investigate the spin depolarization mechanism in a series of L-MHPs with different A-site cations: (Hexa)_2_MAPb_2_I_7_, (Hexa)_2_FAPb_2_I_7_ and (Hexa)_2_CsPb_2_I_7_. All samples show a peculiar trend of longer spin lifetime at low temperatures for excitation with excess photon energy. Moreover, upon resonant excitation at low temperatures, all samples show a monoexponential spin lifetime of ≈200 fs, that we attribute to pure exciton spin relaxation. However, this monoexponential regime persists up to different temperatures for each composition: ∼64 K for MA^+^, ∼77 K for FA^+^, and ∼130 K for Cs^+^. We assign the onset of a biexponential regime to the formation of exciton polarons with distinct spin relaxation dynamics and take the threshold temperature for this regime as a measure of the polaron formation barrier. These temperature thresholds follow the opposite trend of the polarities of MA^+^, FA^+^ and Cs^+^, suggesting that the larger the polarity of the cation, the lower the barrier for polaron formation, indicating that the larger dipole moment helps stabilize the lattice distortion associated with polaron formation. These experimental findings agree with first-principles numerical calculations, which indicate a higher polaron stabilization barrier with increasing dipole moment of the cation. Although A-site cations indirectly affect the electronic structure of lead-halide perovskites by modulating the extent of octahedra tilting in relation to their increasing size,^51^ thus affecting the band gap and carrier effective masses, it is the polarity that fully correlates with our experimental observations. MA is indeed expected to be more polar than FA and Cs. So, both effects, structural/electronic and electrostatic, are expected to interplay in defining the fate of charge carriers. Our results show how both material composition and excitation wavelength can be exploited for tuning the spin lifetime in L-MHPs, which offers a general handle for the realization of optimized materials for opto-spintronic devices.

## Supplementary Material



## Data Availability

The data underlying this study are openly available in Zenodo at https://doi.org/10.5281/zenodo.15193607.

## Notes

Details on the material synthesis, ultrafast spectroscopy and Faraday rotation experiments, computational calculations, and supporting figures and tables (PDF)

## References

[1] Grancini G., Nazeeruddin M. K. (2019). Dimensional Tailoring of Hybrid Perovskites for Photovoltaics. Nat. Rev. Mater..

[2] Blancon J.-C., Even J., Stoumpos C. C., Kanatzidis M. G., Mohite A. D. (2020). Semiconductor Physics of Organic-Inorganic 2D Halide
Perovskites. Nat. Nanotechnol..

[3] Blancon J.-C., Stier A. V., Tsai H., Nie W., Stoumpos C. C., Traore B., Pedesseau L., Kepenekian M., Katsutani F., Noe G. T. (2018). Scaling Law for Excitons
in 2D Perovskite Quantum Wells. Nat. Commun..

[4] Mauck C. M., Tisdale W. A. (2019). Excitons in 2D Organic-Inorganic
Halide Perovskites. Trends Chem..

[5] Straus D. B., Hurtado Parra S., Iotov N., Zhao Q., Gau M. R., Carroll P. J., Kikkawa J. M., Kagan C. R. (2020). Tailoring Hot Exciton
Dynamics in 2D Hybrid Perovskites through Cation Modification. ACS Nano.

[6] Kopteva N. E., Yakovlev D. R., Yalcin E., Kalitukha I. V., Akimov I. A., Nestoklon M. O., Turedi B., Hordiichuk O., Dirin D. N., Kovalenko M. V. (2025). Effect
of Crystal Symmetry of Lead
Halide Perovskites on the Optical Orientation of Excitons. Adv. Sci..

[7] Straus D. B., Kagan C. R. (2018). Electrons, Excitons, and Phonons in Two-Dimensional
Hybrid Perovskites: Connecting Structural, Optical, and Electronic
Properties. J. Phys. Chem. Lett..

[8] Faini F., Asensio Y., Visentin F., Olano-Vegas L., Hörmann M., Hueso L. E., Cerullo G., Camargo F. V. A., Martín-García B., Grancini G. (2025). Imaging Ultrafast Charge
Transfer at Low-Dimensional Lead Halide Perovskite Heterostructures. ACS Energy Lett..

[9] Pimenta M. A., Owen J. S., Guo Y., Brus L. E., Egger D. A., Kronik L., Kanatzidis M. G., Rappe A. M., Hull T., Yaffe O., Zheng F., Stoumpos C. C., Heinz T. F., Tan L. Z. (2017). Local Polar Fluctuations
in Lead Halide Perovskite
Crystals. Phys. Rev. Lett..

[10] Buizza L. R. V., Herz L. M. (2021). Polarons and Charge
Localization in Metal-halide Semiconductors
for Photovoltaic and Light-emitting Devices. Adv. Mater..

[11] Hurtado
Parra S., Straus D. B., Fichera B. T., Iotov N., Kagan C. R., Kikkawa J. M. (2022). Large Exciton Polaron Formation in
2D Hybrid Perovskites via Time-Resolved Photoluminescence. ACS Nano.

[12] Srimath
Kandada A. R., Silva C. (2020). Exciton Polarons in Two-Dimensional
Hybrid Metal-Halide Perovskites. J. Phys. Chem.
Lett..

[13] Giovanni D., Ma H., Chua J., Grätzel M., Ramesh R., Mhaisalkar S., Mathews N., Sum T. C. (2015). Highly
Spin-Polarized Carrier Dynamics
and Ultralarge Photoinduced Magnetization in CH3NH3PbI3 Perovskite
Thin Films. Nano Lett..

[14] Li W., Zhou L., Prezhdo O. V., Akimov A. V. (2018). Spin-Orbit Interactions
Greatly Accelerate Nonradiative Dynamics in Lead Halide Perovskites. ACS Energy Lett..

[15] Righetto M., Giovanni D., Lim S. S., Sum T. C. (2021). The Photophysics
of Ruddlesden-Popper Perovskites: A Tale of Energy, Charges, and Spins. Appl. Phys. Rev..

[16] Kopteva N. E., Yakovlev D. R., Yalcin E., Akimov I. A., Nestoklon M. O., Glazov M. M., Kotur M., Kudlacik D., Zhukov E. A., Kirstein E., Hordiichuk O., Dirin D. N., Kovalenko M. V., Bayer M. (2024). Highly-Polarized Emission
Provided by Giant Optical Orientation of
Exciton Spins in Lead Halide Perovskite Crystals. Adv. Sci..

[17] Todd S. B., Riley D. B., Binai-Motlagh A., Clegg C., Ramachandran A., March S. A., Hoffman J. M., Hill I. G., Stoumpos C. C., Kanatzidis M. G., Yu Z. G., Hall K. C. (2019). Detection of Rashba
Spin Splitting in 2D Organic-Inorganic Perovskite via Precessional
Carrier Spin Relaxation. APL Mater..

[18] Haque M. A., Beard M. (2025). Spin Effects in Metal
Halide Perovskite Semiconductors. Nanoscale.

[19] Chen Z., Dong G., Qiu J. (2021). Ultrafast
Pump-probe Spectroscopy—a
Powerful Tool for Tracking Spin-quantum Dynamics in Metal Halide Perovskites. Adv. Quantum Technol..

[20] Liao K., Hu X., Cheng Y., Yu Z., Xue Y., Chen Y., Gong Q. (2019). Spintronics of Hybrid Organic-Inorganic Perovskites: Miraculous Basis
of Integrated Optoelectronic Devices. Adv. Opt.
Mater..

[21] Lu Y., Wang Q., Han L., Zhao Y., He Z., Song W., Song C., Miao Z. (2024). Spintronic Phenomena
and Applications in Hybrid Organic-Inorganic Perovskites. Adv. Funct. Mater..

[22] Privitera A., Righetto M., Cacialli F., Riede M. K. (2021). Perspectives of
Organic and Perovskite-based Spintronics. Adv.
Opt. Mater..

[23] Lee C. U., Lee H., Jeong C. S., Ma S., Jang G., Park Y. S., Yun J., Lee J., Son J., Jeong W., Yang S., Park J. H., Woo K., Moon J. (2024). Enhanced Stability
of Spin-Dependent Chiral 2D Perovskite Embedded PV-Biased Anode via
Cross-Linking Strategy. ACS Energy Lett..

[24] Chen D., Tang B., Sergeev A. A., Wu Y., Liu H., Zhu D., Hu S., Wong K. S., Yip H. L., Rogach A. L. (2025). Green Spin
Light-Emitting Diodes Enabled by Perovskite Nanocrystals in Situ Modified
with Chiral Ligands. ACS Energy Lett..

[25] Lu H., Wang J., Xiao C., Pan X., Chen X., Brunecky R., Berry J. J., Zhu K., Beard M. C., Vardeny Z. V. (2019). Spin-Dependent Charge Transport through
2D Chiral Hybrid
Lead-Iodide Perovskites. Sci. Adv..

[26] Dong Y., Hautzinger M. P., Haque M. A., Beard M. C. (2025). Chirality-Induced
Spin Selectivity in Hybrid Organic-Inorganic Perovskite Semiconductors. Annu. Rev. Phys. Chem..

[27] Kim Y. H., Zhai Y., Lu H., Pan X., Xiao C., Gaulding E. A., Harvey S. P., Berry J. J., Vardeny Z. V., Luther J. M., Beard M. C. (2021). Chiral-Induced Spin Selectivity Enables
a Room-Temperature Spin Light-Emitting Diode. Science (80-.).

[28] Li S., Xu X., Kocoj C. A., Zhou C., Li Y., Chen D., Bennett J. A., Liu S., Quan L., Sarker S., Liu M., Qiu D. Y., Guo P. (2024). Large Exchange-Driven Intrinsic Circular
Dichroism of a Chiral 2D Hybrid Perovskite. Nat. Commun..

[29] Sercel P. C., Hautzinger M. P., Song R., Blum V., Beard M. C. (2025). Optical
Activity of Chiral Excitons. Adv. Mater..

[30] Chen X., Lu H., Li Z., Zhai Y., Ndione P. F., Berry J. J., Zhu K., Yang Y., Beard M. C. (2018). Impact of Layer Thickness on the
Charge Carrier and Spin Coherence Lifetime in Two-Dimensional Layered
Perovskite Single Crystals. ACS Energy Lett..

[31] Chen X., Lu H., Wang K., Zhai Y., Lunin V., Sercel P. C., Beard M. C. (2021). Tuning Spin-Polarized Lifetime in Two-Dimensional Metal-Halide
Perovskite through Exciton Binding Energy. J.
Am. Chem. Soc..

[32] Zhukov E. A., Yakovlev D. R., Kirstein E., Kopteva N. E., Hordiichuk O., Kovalenko M. V., Bayer M. (2025). Coherent Spin Dynamics of Electrons
and Holes Photogenerated with Large Kinetic Energy in Lead Halide
Perovskite Crystals. ACS Photonics.

[33] Grisard S., Trifonov A. V., Hahn T., Kuhn T., Hordiichuk O., Kovalenko M. V., Yakovlev D. R., Bayer M., Akimov I. A. (2024). Spin-Dependent
Exciton-Exciton Interactions in a Mixed Lead Halide Perovskite Crystal. ACS Photonics.

[34] Feng S., Badalis C. J., Gloor C. J., Zhong X., Gan Z., You W., Moran A. M. (2025). Nonlinear
Optical Signatures of Spin Relaxation in
2D Perovskites. J. Chem. Phys..

[35] Li Y., Luo X., Liu Y., Lu X., Wu K. (2020). Size- And Composition-Dependent
Exciton Spin Relaxation in Lead Halide Perovskite Quantum Dots. ACS Energy Lett..

[36] Liang W., Li Y., Xiang D., Han Y., Jiang Q., Zhang W., Wu K. (2021). Efficient Optical Orientation
and Slow Spin Relaxation in Lead-Free
CsSnBr 3Perovskite Nanocrystals. ACS Energy
Lett..

[37] Bourelle S. A., Camargo F. V. A., Ghosh S., Neumann T., Van De Goor T. W. J., Shivanna R., Winkler T., Cerullo G., Deschler F. (2022). Optical Control
of Exciton Spin Dynamics in Layered Metal Halide Perovskites via Polaronic
State Formation. Nat. Commun..

[38] Abdel-Baki K., Boitier F., Diab H., Lanty G., Jemli K., Lédée F., Garrot D., Deleporte E., Lauret J.-S. (2016). Exciton Dynamics
and Non-Linearities in Two-Dimensional
Hybrid Organic Perovskites. J. Appl. Phys..

[39] Yin J., Maity P., Naphade R., Cheng B., He J.-H., Bakr O. M., Brédas J.-L., Mohammed O. F. (2019). Tuning Hot Carrier
Cooling Dynamics by Dielectric Confinement in Two-Dimensional Hybrid
Perovskite Crystals. ACS Nano.

[40] Burgos-Caminal A., Socie E., Bouduban M. E. F., Moser J.-E. (2020). Exciton and Carrier
Dynamics in Two-Dimensional Perovskites. J.
Phys. Chem. Lett..

[41] Bourelle S. A., Shivanna R., Camargo F. V. A., Ghosh S., Gillett A. J., Senanayak S. P., Feldmann S., Eyre L., Ashoka A., Van De Goor T. W. J., Abolins H., Winkler T., Cerullo G., Friend R. H., Deschler F. (2020). How Exciton Interactions Control
Spin-Depolarization in Layered Hybrid Perovskites. Nano Lett..

[42] Ambrosio F., Meggiolaro D., Mosconi E., De Angelis F. (2019). Charge Localization,
Stabilization, and Hopping in Lead Halide Perovskites: Competition
between Polaron Stabilization and Cation Disorder. ACS Energy Lett..

[43] Mahata A., Meggiolaro D., De Angelis F. (2019). From Large to Small Polarons in Lead,
Tin, and Mixed Lead-Tin Halide Perovskites. J. Phys. Chem. Lett..

[44] Stoumpos C. C., Cao D. H., Clark D. J., Young J., Rondinelli J. M., Jang J. I., Hupp J. T., Kanatzidis M. G. (2016). Ruddlesden-Popper
Hybrid Lead Iodide Perovskite 2D Homologous Semiconductors. Chem. Mater..

[45] Ambrosio F., Meggiolaro D., Mosconi E., De Angelis F. (2020). Charge Localization
and Trapping at Surfaces in Lead-Iodide Perovskites: The Role of Polarons
and Defects. ACS Energy Lett..

[46] Meggiolaro D., Ambrosio F., Mosconi E., Mahata A., De Angelis F. (2020). Polarons in
Metal Halide Perovskites. Adv. Energy Mater..

[47] Ambrosio F., Wiktor J., De Angelis F., Pasquarello A. (2018). Origin of
Low Electron-Hole Recombination Rate in Metal Halide Perovskites. Energy Environ. Sci..

[48] Fu J., Ramesh S., Melvin Lim J. W., Sum T. C. (2023). Carriers, Quasi-Particles,
and Collective Excitations in Halide Perovskites. Chem. Rev..

[49] Pérez-Osorio M. A., Lin Q., Phillips R. T., Milot R. L., Herz L. M., Johnston M. B., Giustino F. (2018). Raman Spectrum
of the Organic-Inorganic Halide Perovskite
CH3NH3PbI3 from First Principles and High-Resolution Low-Temperature
Raman Measurements. J. Phys. Chem. C.

[50] Pérez-Osorio M. A., Milot R. L., Filip M. R., Patel J. B., Herz L. M., Johnston M. B., Giustino F. (2015). Vibrational Properties of the Organic-Inorganic
Halide Perovskite CH3NH3PbI3 from Theory and Experiment: Factor Group
Analysis, First-Principles Calculations, and Low-Temperature Infrared
Spectra. J. Phys. Chem. C.

[51] Amat A., Mosconi E., Ronca E., Quarti C., Umari P., Nazeeruddin M. K., Grätzel M., De Angelis F. (2014). Cation-Induced
Band-Gap Tuning in Organohalide Perovskites: Interplay of Spin-Orbit
Coupling and Octahedra Tilting. Nano Lett..

