# Supplementary material for: Cation Tuning of Polaron Barriers in Layered Perovskites for Optical Spin Lifetime Control
Source: ACS Energy Lett. 2025 Aug 31;10(9):4636–43. doi: 10.1021/acsenergylett.5c01236 (PMC12442098; doi:10.1021/acsenergylett.5c01236)
Supplement: Supplementary file 1 [file nz5c01236_si_001.pdf]

Supporting Information

Cation Tuning of Polaron Barriers in Layered Perovskites for Optical Spin Lifetime Control

Valentino Romano[1], Martin Hörmann[1], Anna Stadlbauer[2,3], Edoardo Mosconi[4], Luca Gregori[4,5], Filippo De Angelis[4,5,6], Felix Deschler[3,], Giulio Cerullo[1,7], Franco V. A. Camargo[7,*]*

[1]Physics Department, Politecnico di Milano, Piazza Leonardo da Vinci 32, 20133, Milano, Italy

[2]Walter-Schottky-Institute, Physics Department, Technical University Munich, 85748, Germany

[3]Institute for Physical Chemistry, Heidelberg University, Im Neuenheimer Feld 229, 69120 Heidelberg

[4]Computational Laboratory for Hybrid/Organic Photovoltaics (CLHYO), Istituto CNR di Scienze e Tecnologie Chimiche "Giulio Natta" (CNR-SCITEC), Via Elce di Sotto 8, 06123 Perugia, Italy

[5]Department of Chemistry, Biology and Biotechnology, University of Perugia, 06123, Italy

[6]SKKU Institute of Energy Science and Technology (SIEST), Sungkyunkwan University, Suwon 440-746, South Korea

[7]Istituto di Fotonica e Nanotecnologie, Consiglio Nazionale delle Ricerche, Piazza Leonardo da Vinci 32, 20133, Milano, Italy

1. Material synthesis

For the fabrication of spin-coated thin film samples, we used suitable combinations of the precursor powders of Hexylammonium Iodide ($C_6H_{16}IN$), Methylammonium Iodide (CH_6IN), Formamidinium Iodide (CH_5IN_2), Cesium Iodide (CsI) and Lead Iodide (PbI_2), which were dissolved in 2 ml Dimethylformamid (DMF) to achieve three 0.1 molar solutions. The solutions

were mixed with stirring bars at 300 rpm while being heated on a 90°C hotplate for about two hours.

Glass substrates with 1 mm thickness were cut in a suitable size of about 1.5 cm^2 area. They were cleaned for each 5 min in an ultrasonic bath in acetone and isopropanol. Afterwards, the were rinsed with isopropanol, dried using a nitrogen nozzle and then cleaned in an oxygen plasma for 5 min at 200 W. The cleaned samples were brought to a glovebox with nitrogen atmosphere, where the precursor solutions were still on the hotplate.

Next, the precursor solutions were cooled down by placing them on a metal plate. Then they were filtered through a PTFE-syringe filter with a pore diameter of 0.2 µm. On each cleaned glass substrate, 80 µl of the solution were spin-coated with 5000 rpm for 25 sec with a ramp of 1500 rpm. As quickly as possible, the spin-coated films were put on a 140°C warm hotplate and kept there for three minutes. Finally, the samples were taken from the hotplate and stored in the dark under nitrogen atmosphere.

2. Time resolved Faraday rotation and absorption measurements

Both TRFR and TA measurements were performed using a regeneratively amplified Ti:Sapphire laser (Coherent Libra), operating at 2 kHz, with a fundamental emission at 1.549 eV (800 nm). For TRFR measurements pump and probe pulses, with a bandwidth of 40 meV full-width-half-maximum and a duration of 70 fs, were generated through home-built optical parametric amplifiers (OPAs). The pump beam was circularly polarized by a quarter waveplate mounted just before the sample, while the probe beam was kept with a linear polarization. A chopper at 1 kHz was used along the pump path and the probe was detected using Wollaston prism and two balanced photodiodes: the prism is used to split the probe into two orthogonally polarized components, each one sent on one of the balanced photodiodes so that both photodiodes read the same signal when the probe beam arrives before the pump beam (i.e., at negative pump-probe delays). In this way, common-mode intensity fluctuations of the probe beam are suppressed. The TRFR signal, which ranges between 10^{-5} and 10^{-7}, is detected using a lock-in amplifier.

For the TA measurements, the fundamental wavelength of the Ti:Sapphire laser is used to generate a supercontinuum (between 2.583 and 1.771 eV) on a sapphire crystal (probe) and a narrowband (40 meV) pump beam through an OPA. After the sample, the transmitted probe beam is sent to a

monochromator and its spectrum is detected with a CCD array. The measured quantity is $\Delta T/T = (T_{on} - T_{off})/T_{off}$, where T_{on} and T_{off} are the intensities of the transmitted probe with the pump unblocked or blocked, respectively, by an optical chopper (at a frequency of 1 kHz). The time delay between pump and probe is changed with an automated delay stage that can reach up to 1.25 ns.

Low temperature measurements were performed with a continuous flow cryostat (MicrostatHires, Oxford Instruments) using a transfer-line connected to a helium dewar.

3. Computational details

Density Functional Theory (DFT) calculations were performed starting from the experimental monoclinic phase of $(HexA)_2MAPb_2I_7$ as reported in reference 1 using the Quantum Espresso software package.[2,3] Both lattice parameters and ionic positions were optimized for systems incorporating various "3D" cations, namely MA, FA and Cs. The optimized lattice parameters are provided in **table S1**. Calculations employed the GGA-PBE functional[4] with norm-conserving (NC) pseudopotentials from pseudo dojo repository,[5] (shells explicitly included in calculations: I 5s, 5p; N, C 2s, 2p; O 2s 2p; H 1s; Pb 6s, 6p, 5d) and a cutoff energy of 80 Ryd (320 Ryd on the charge density). Due to the size of the cells, we optimized all the structures utilizing a 4 x 4 x 1 K-points grid centered in the Brillouin Zone.

To investigate hole polarons, 2 × 2 × 1 supercells were constructed based on the optimized unit cell. Structural relaxations for both the neutral and positively charged states were conducted using the hybrid PBE0 functional,[6] implemented within the CP2K software suite.[7] Hybrid functional calculations used the original fraction of Fock exchange (α = 0.25) and included dispersion interactions via the Grimme D3 scheme.[8]

Core–valence interactions were treated with Goedecker–Teter–Hutter pseudopotentials,[9] while wavefunctions were expanded using double-ζ polarized basis sets and a density cutoff of 400 Ry. The auxiliary density matrix method with the cFIT auxiliary basis set was employed to accelerate hybrid functional computations.[10] Although spin-orbit coupling (SOC) significantly affects the conduction band edge in lead-based perovskites[11,12], its impact on the valence band, relevant for hole polaron formation, is modest[13]. Prior studies have shown that SOC has negligible influence

on polaron localization[14] and only minor effects on stabilization energies[15]. Therefore, SOC is not expected to significantly affect our polaron energy estimates based on total energy differences, allowing us to reduce the overall computational burden.

4. Supporting Figures

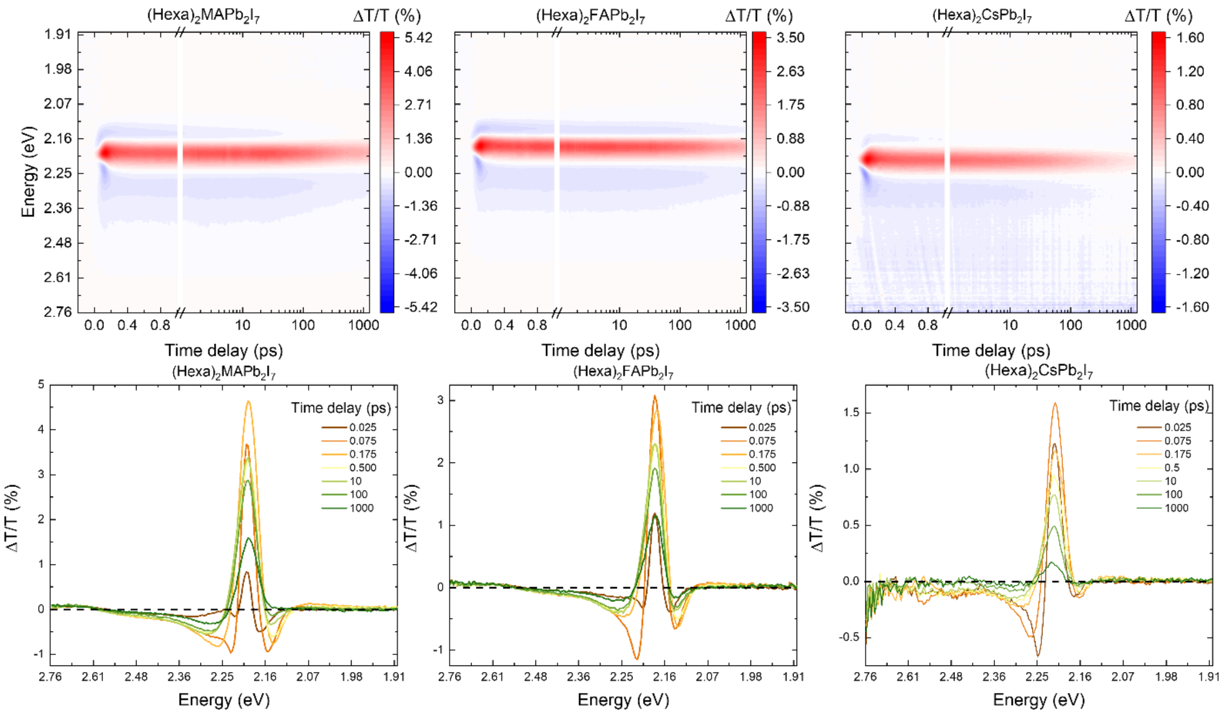

Figure S1: Transient absorption characterization of $(Hexa)_2MAPb_2I_7$, $(Hexa)_2FAPb_2I_7$ and $(Hexa)_2CsPb_2I_7$ measured at 290 K and with excitation in resonance with the optical bandgaps of the materials. Both maps (in the first row) and spectra (in the second row) show no sign of parasitic contributions coming from $n \neq 2$ phases, confirming the good quality of the investigated samples.

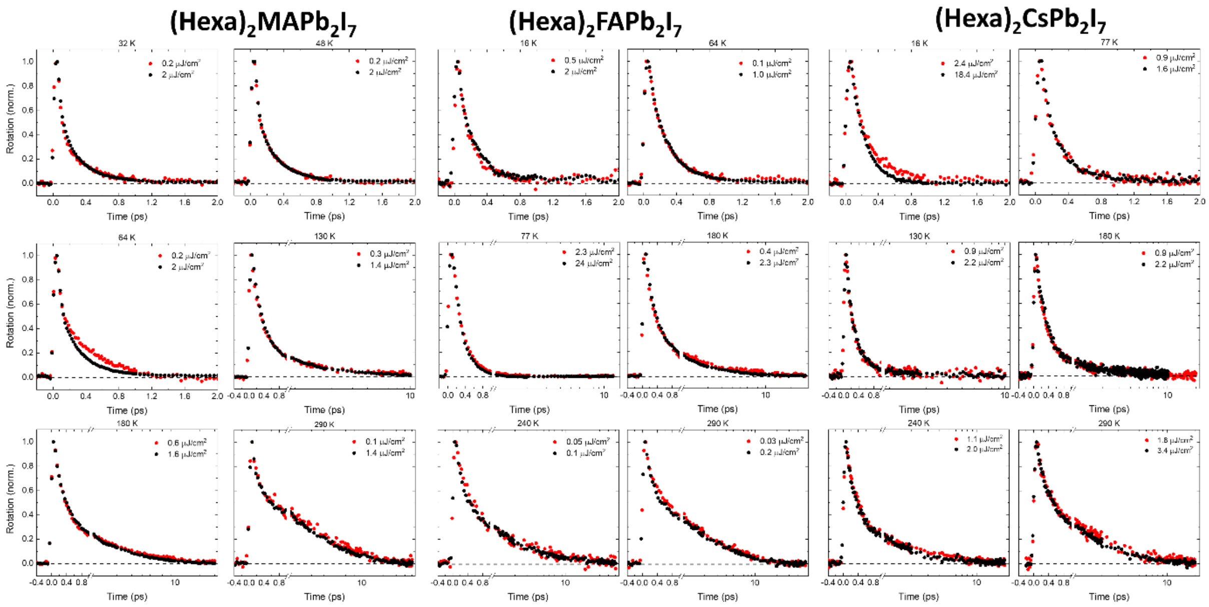

Figure S2. Confirmation that the data presented in Figure 3 of the main text corresponds to the monomolecular regime. The coincidence of the TRFR kinetics at two different fluences establishes that both are in the monomolecular regime. The only two exceptions are MA at 64 K and Cs at 16 K, but in both these cases the low fluence at the next highest temperature ensures that the monomolecular regime still holds.

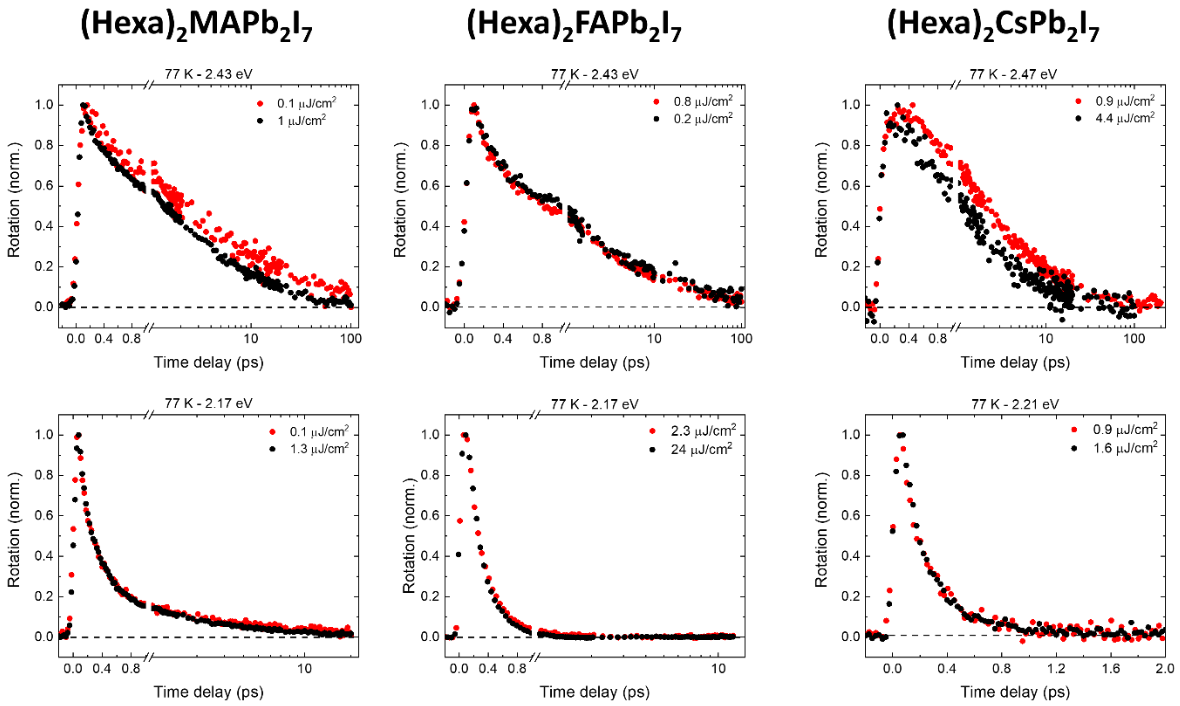

Figure S3. Data of Figure 2 of the main text including a higher fluence. We note that for MA and Cs following excitation with excess energy the coincidence is not perfect, indicating some many-body kinetics at the higher fluence. Although this prevents us from fully excluding the presence of nonlinear kinetics at the lower fluence, the data still unequivocally demonstrates that excitation with excess energy at 77 K leads to a hundred-fold increase in the spin lifetime.

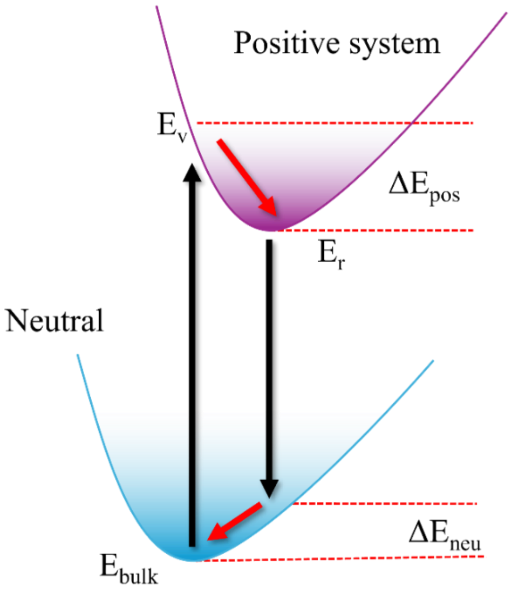

Figure S4: Scheme for the hole-polaron formation mechanism.

System	Lattice parameters (Å)	
$(Hexa)_2MAPb_2I_7$	a = 44.29	α = 90°
	b = 8.612	β = 100.22°
	c = 8.553	γ = 90°
$(Hexa)_2FAPb_2I_7$	a = 44.03	α = 90°
	b = 8.695	β = 100.24°
	c = 8.532	γ = 90°
$(Hexa)_2CsPb_2I_7$	a = 44.40	α = 90°
	b = 8.641	β = 100.21°
	c = 8.536	γ = 90°

Table S1: Optimized lattice parameters and angles for the three different cations at PBE-D3 level of theory.

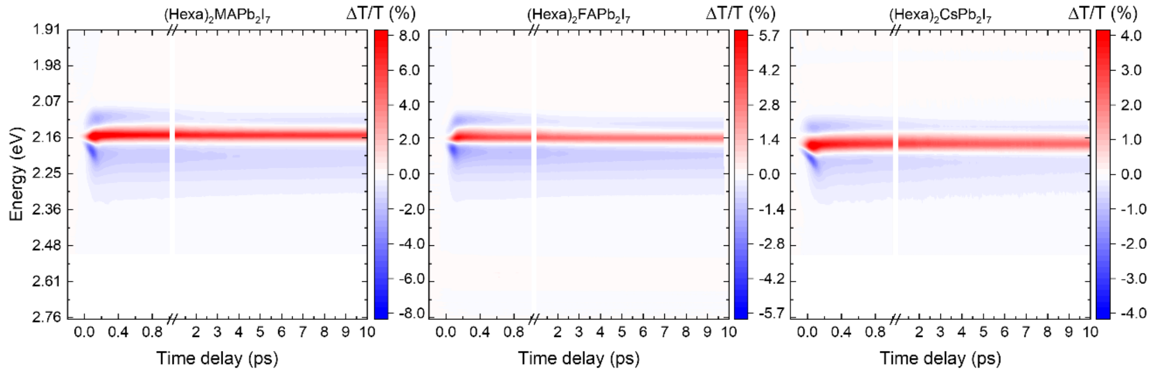

Figure S5: Transient absorption maps of $(Hexa)_2MAPb_2I_7$, $(Hexa)_2FAPb_2I_7$ and $(Hexa)_2CsPb_2I_7$ measured at 77K and with excitation in resonance with the optical bandgaps of the materials.

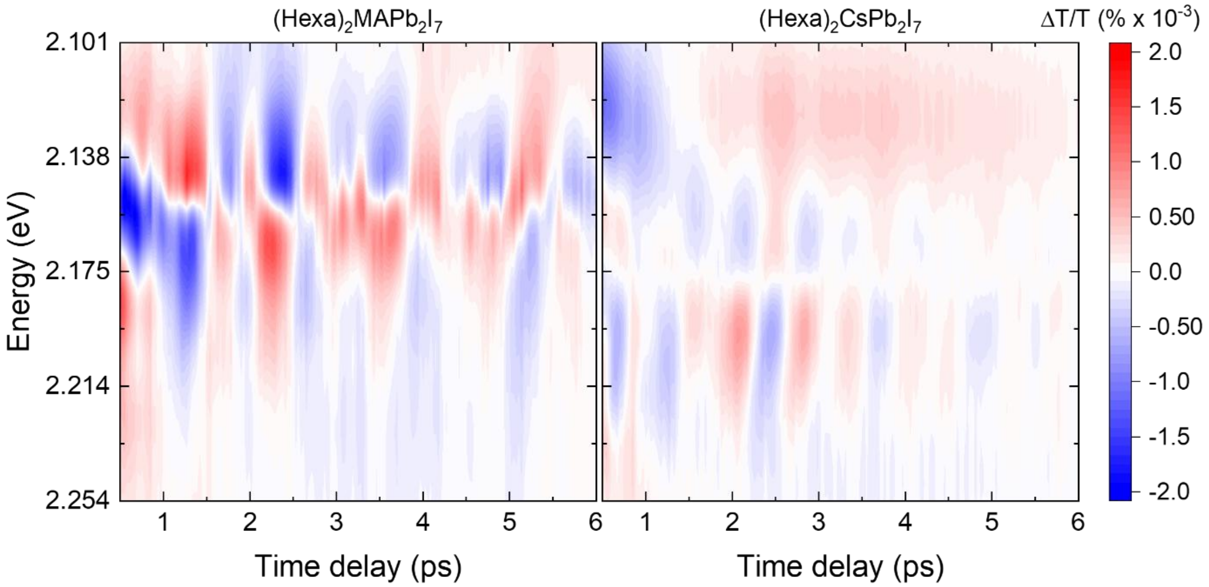

Figure S6: Oscillatory component of the transient absorption signal of $(Hexa)_2MAPb_2I_7$ and $(Hexa)_2FAPb_2I_7$.

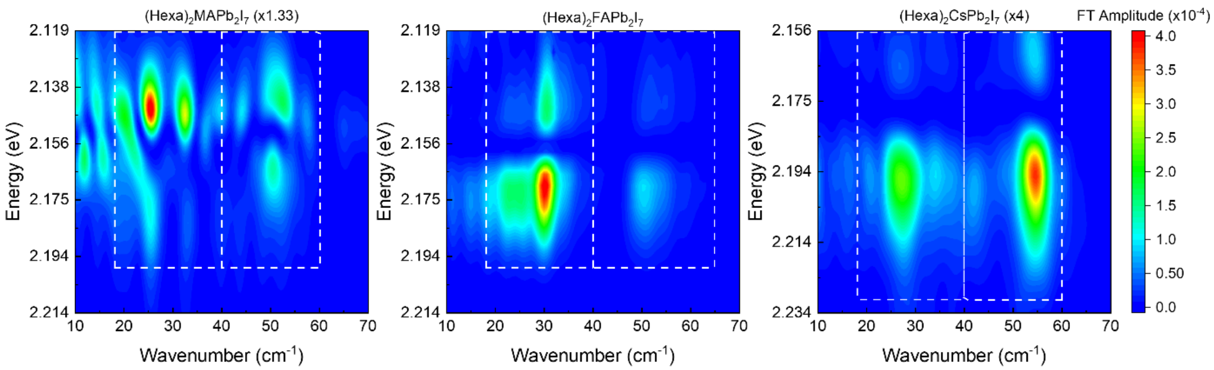

Figure S7: Power spectra of the investigated samples showing as dashed rectangles the ranges chosen for the calculation of the integral of the power spectra. Wavelength: 2.119-2.198 eV (for $(Hexa)_2MAPb_2I_7$ and $(Hexa)_2FAPb_2I_7$); 2.156-2.230 eV (for $(Hexa)_2CsPb_2I_7$). Wavenumber: 18-40 cm^{-1}; 40-60 cm^{-1}.

5. References

(1) Fu, Y.; Hautzinger, M. P.; Luo, Z.; Wang, F.; Pan, D.; Aristov, M. M.; Guzei, I. A.; Pan, A.; Zhu, X.; Jin, S. Incorporating Large A Cations into Lead Iodide Perovskite Cages: Relaxed Goldschmidt Tolerance Factor and Impact on Exciton-Phonon Interaction. *ACS Cent. Sci.* **2019**, *5* (8), 1377–1386. https://doi.org/10.1021/acscentsci.9b00367.

(2) Giannozzi, P.; Andreussi, O.; Brumme, T.; Bunau, O.; Nardelli, M. B.; Calandra, M.; Car, R.; Cavazzoni, C.; Ceresoli, D.; Cococcioni, M.; Colonna, N.; Carnimeo, I.; Dal Corso, A.; de Gironcoli, S.; Delugas, P.; DiStasio Jr, R. A.; Ferretti, A.; Floris, A.; G, F.; G, F.; Gebauer, R.; Gerstmann, U.; Giustino, F.; Gorni, T.; Jia, J.; Kawamura, M.; Ko, H.-Y.; Kokalj, A.; Kucukbenli, E.; Lazzeri, M.; Marsili, M.; Marzari, N.; Mauri, F.; Nguyen, N. L.; Nguyen, H.-V.; Otero-de-la-Roza, A.; Paulatto, L.; Poncé, S.; Rocca, D.; Sabatini, R.; Santra, B.; Schlipf, M.; Seitsonen, A. P.; Smogunov, A.; Timrov, I.; Thonhauser, T.; Umari, P.; Vast, N.; Wu, X.; Baroni, S. Advanced Capabilities for Materials Modelling with Quantum ESPRESSO. *J. Phys. Condens. matter* **2017**, *29* (46), 465901.

(3) Giannozzi, P.; Baroni, S.; Bonini, N.; Calandra, M.; Car, R.; Cavazzoni, C.; Ceresoli, D.; Chiarotti, G. L.; Cococcioni, M.; Dabo, I.; Dal Corso, A.; de Gironcoli, S.; Fabris, S.; Fratesi, G.; Gebauer, R.; Gerstmann, U.; Gougoussis, C.; Kokalj, A.; Lazzeri, M.; Paolini,

S.; Pasquarello, A.; Paulatto, L.; Sbraccia, C.; Scandolo, S.; Sclauzero, G.; Seitsonen, A. P.; Smogunov, A.; Umari, P.; Wentzcovitch, R. M. QUANTUM ESPRESSO: A Modular and Open-Source Software Project for Quantum Simulations of Materials. *J. Phys. Condens. matter* **2009**, *21* (39), 395502.

(4) Ernzerhof, M.; Scuseria, G. E. Assessment of the Perdew–Burke–Ernzerhof Exchange-Correlation Functional. *J. Chem. Phys.* **1999**, *110* (11), 5029–5036.

(5) Van Setten, M. J.; Giantomassi, M.; Bousquet, E.; Verstraete, M. J.; Hamann, D. R.; Gonze, X.; Rignanese, G.-M. The PseudoDojo: Training and Grading a 85 Element Optimized Norm-Conserving Pseudopotential Table. *Comput. Phys. Commun.* **2018**, *226*, 39–54.

(6) Adamo, C.; Barone, V. Toward Reliable Density Functional Methods without Adjustable Parameters: The PBE0 Model. *J. Chem. Phys.* **1999**, *110* (13), 6158–6170.

(7) Kühne, T. D.; Iannuzzi, M.; Del Ben, M.; Rybkin, V. V; Seewald, P.; Stein, F.; Laino, T.; Khaliullin, R. Z.; Schütt, O.; Schiffmann, F.; Golze, D.; Wilhelm, J.; Chulkov, S.; Hossein, B.-H. M.; Weber, V.; Borstnik, U.; Taillefumier, M.; Andermatt, S.; Holmberg, N.; Shenter, G. K.; Hehn, A.; Bussy, A.; Belleflamme, F.; Tabacchi, G.; Glob, A.; Lass, M.; Bethune, I.; Mundy, C. J.; Plessl, C.; Watkins, M.; VandeVondele, J.; Krack, M.; Hutter, J. CP2K: An Electronic Structure and Molecular Dynamics Software Package-Quickstep: Efficient and Accurate Electronic Structure Calculations. *J. Chem. Phys.* **2020**, *152* (19).

(8) Grimme, S.; Antony, J.; Ehrlich, S.; Krieg, H. A Consistent and Accurate Ab Initio Parametrization of Density Functional Dispersion Correction (DFT-D) for the 94 Elements H-Pu. *J. Chem. Phys.* **2010**, *132* (15).

(9) Goedecker, S.; Teter, M.; Hutter, J. Separable Dual-Space Gaussian Pseudopotentials. *Phys. Rev. B* **1996**, *54* (3), 1703.

(10) Guidon, M.; Hutter, J.; VandeVondele, J. Auxiliary Density Matrix Methods for Hartree−Fock Exchange Calculations. *J. Chem. Theory Comput.* **2010**, *6* (8), 2348–2364. https://doi.org/10.1021/ct1002225.

(11) Even, J.; Pedesseau, L.; Jancu, J. M.; Katan, C. Importance of Spin-Orbit Coupling in

Hybrid Organic/Inorganic Perovskites for Photovoltaic Applications. *J. Phys. Chem. Lett.* **2013**, *4* (17), 2999–3005. https://doi.org/10.1021/jz401532q.

(12) Umari, P.; Mosconi, E.; De Angelis, F. Relativistic GW Calculations on CH3 NH3 PbI 3 and CH3 NH3 SnI3 Perovskites for Solar Cell Applications. *Sci. Rep.* **2014**, *4*, 4467. https://doi.org/10.1038/srep04467.

(13) Meggiolaro, D.; De Angelis, F. First-Principles Modeling of Defects in Lead Halide Perovskites: Best Practices and Open Issues. *ACS Energy Lett.* **2018**, *3* (9), 2206–2222. https://doi.org/10.1021/acsenergylett.8b01212.

(14) Ambrosio, F.; Wiktor, J.; De Angelis, F.; Pasquarello, A. Origin of Low Electron–Hole Recombination Rate in Metal Halide Perovskites. *Energy Environ. Sci.* **2018**, *11* (1), 101–105.

(15) Mahata, A.; Meggiolaro, D.; De Angelis, F. From Large to Small Polarons in Lead, Tin, and Mixed Lead-Tin Halide Perovskites. *J. Phys. Chem. Lett.* **2019**, *10* (8), 1790–1798. https://doi.org/10.1021/acs.jpclett.9b00422.
